# Aging Brain: Prevention of Oxidative Stress by Vitamin E and Exercise

**DOI:** 10.1100/tsw.2009.46

**Published:** 2009-05-22

**Authors:** Sambe Asha Devi

**Affiliations:** Laboratory of Gerontology, Department of Zoology, Bangalore University, Bangalore 560 056, India

**Keywords:** aging, acetylcholine esterase, cerebral cortex, cognition, exercise, healthy brain, oxidative stress, vitamin E

## Abstract

With aging, the brain undergoes neuronal loss in many areas. Although the loss of cells in the cerebral cortex, in particular the frontal cortex, has been recognized with aging, the influence of synaptic losses has a larger impact on cognitive decline. Much of the recent research on animals, as well as humans, has been aimed at slowing the cognitive decline through enrichment, and it has been found that the key factors are antioxidants and exercise. Several reports support the concept that regular supplementation of vitamin E and physical activity from as early as middle age can slow the cognitive decline observed during the later years. A few studies have also suggested that exercise is analogous to acetylcholine esterase inhibitors that are also used extensively to treat cognitive impairment and dementia in Alzheimer's disease. In addition, reports also support that vitamin E and exercise may act synergistically to overcome free radical injury and oxidative stress in the aging brain.

## INTRODUCTION

The last 2 decades have witnessed tremendous efforts by gerontologists to find means for a healthy life and healthy brain aging. Advanced age is associated with a greater susceptibility of the brain to free radicals, and overcoming the ravages of these free radicals has been a challenge. Studies that deal with counteracting mechanisms speak of the work of numerous researchers to evaluate these mechanisms, not only in the case of advanced age that is accompanied with memory deficit, but also of the cholinergic-related neuropathological diseases such as Alzheimer's disease (AD). A cardinal feature of brain aging is the slowing of cognitive processing in the cerebral cortex (CC) and hippocampus (HC), the sites for learning and memory.

In fact, it is the overlapping of several diseases that occur with aging in humans that overshadows our attempts to link the decline in mental performance to normal aging *per se*. However, laboratory animals have always assisted us in differentiating between healthy and pathological aging. Animal models are inevitably used owing to the limitations of human subjects involving individual differences. Of the large number of studies conducted on brain enrichment, the studies that encompass learning and memory have greater significance to humans, and have become a focus of basic science and pharmaceutical research.

This present article reviews the recent progress made in our laboratory and others in slowing brain aging in animals and humans. This review, although not comprehensive, will focus on two strategies, physical exercise and vitamin E, as enriching factors for overcoming oxidative damage in the normal aging of the brain.

## FREE RADICALS AND OXIDATIVE DAMAGE

The generation of free radicals leading to oxidative modifications with aging results in many age-related disorders[1]. Age-dependent oxidative stress (OS) has been demonstrated in *ex vivo* brain slices by oxygen radical–dependent chemiluminescent signals and reactive oxygen signaling as being responsible for the onset of aging in mammals and birds[2]. Free radicals that cause OS modify learning and memory functions in the rat, and it is the CC and HC that are the first regions to undergo degenerative aging[3]. More importantly, OS is a major risk factor that leads to increased lipid peroxidation (LPO) and reduced antioxidant defense in the aging brain. The extent of oxidative damage caused by reactive oxygen species (ROS) is identified by two markers, namely, protein oxidation relating to changes in the levels of protein carbonyl (PrC), protein sulphydryl (P-SH), and advanced protein oxidation products (AOPP), and of LPO to lipofuscin (LF) and thiobarbituric acid substances (TBARS). A positive correlation is seen between age and the above two markers in young and old rats [4]. Reports on neurodegeneration as a consequence of lipid and protein oxidations are also evident in lower animals, such as fruit flies and nematodes[5]. Studies on the primary cultures of neonatal cerebral cortical cells of mice exhibit increase in the autofluorescent LF-like substances with aging *in vitro*[6]. Benavides and colleagues[7] demonstrated a progressive accumulation of neuronal LF, an autofluorescent substance in the human CC right from the first to ninth decade of life.

Despite the fact that the need for substrates such as glucose in the older rat brain is no different from the younger, the older has a significantly low influx of the substrate and is more susceptible to stress than the younger. Further, lowered RNA level in CC in the older is suggestive of a reduced rate of protein synthesis[8]. However, a more important concern in older human subjects is physiological changes in the brain that lead to memory loss and learning skills. Although this cannot be linked to memory loss related to diseased conditions such as AD, the fact that aging itself is a potent risk factor in the evolution of the disease[9,10] cannot be overlooked. Further, AD is the debilitating effect of an imbalance between pro- and antioxidants. In addition, it is noticeable that with age, the geometric incidence of AD is associated with insufficient amounts of acetylcholine (ACh). Researchers have identified a transitory state referred to as mild cognitive impairment (MCI) between normal aging and that associated with AD.

Learning, especially of the spatial type, in aged rats is impaired and is accounted for by a decline in the circuitry and plasticity of regions like the HC[11]. Until the early 1990s, neuroscientists, based on experimental evidence, believed that humans and animals were born with all the required number of neurons that would last their lifetimes. In 1998, Fred Gage (a neuroscientist at the Salk Institute in La Jolla, California) published his studies identifying the formation of new neurons in the adult HC that, along with the cortex, is associated with memory, although the function of these newly formed cells were improperly defined at that time. The production of new neurons in the HC slows dramatically by middle age in rats, the equivalent of 50–55 years in humans. In the aged, the decline in cognitive ability is related to the concomitant impairment of cortical cholinergic neurons. Today, several lines of research have produced convincing evidence to suggest that the mental decline that occurs with aging is largely due to the loss of synaptic connections rather than loss of neurons. For instance, oxidative damage to the synaptic regions of the CC besides HC results in a decline in cognitive functions[12], with the synaptic site being more vulnerable to OS than other sites in the brain[13].

## VITAMIN E AND THE AGING BRAIN

The CC, an area associated with higher cognitive processing, is receptive to various types of environmental enrichments. This suggests that cognitive stimulation in the aged might protect them against dementia. The CC is known to be an ever-changing region, with its micro architecture, and is influenced by experiences even before birth and is responsive to enrichment throughout life[14]. Interestingly, rat cells have new neurons that develop in the adult dentate gyrus, a site for recent memory processing in the HC. The CC has efficient built-in mechanisms to overcome the toxicity of free radicals and this defense is overwhelmed by the aging process. However, the issue at hand is to focus on the influence of exogenous antioxidants, such as vitamin E, in overcoming OS and, more critically, the attempts made for improving the weakened cognitive ability in the aged. Similar to AD, an early loss of cognitive decline and loss of cholinergic neurons in the basal forebrain of humans as well as of a mouse model of Down's syndrome (DS), Ts65Dn has been reported by Lockrow et al.[15]. Further, the scientists demonstrated that vitamin E can delay the onset of cognitive decline and loss of neurons in the same model. Similar studies have indicated that vitamin E can only slow the decline of certain cognitive abilities other than memory, but has no net effect in delaying the progression of AD[16].

In fact, vitamin E includes tocopherols and tocotrienols, with the former being the active form in tissues and is referred to as α-tocopherol (αT). An interesting fact is that the brain possesses mechanisms for greater uptake as well as retention of αT compared to other tissues and is aided through tocopherol transfer protein (TTP). TTP is also essential for maintaining vitamin E levels in the brain[17]. Vitamin E is important due to its ability to detoxify free radicals, such as hydroxyl, superoxide, and peroxyl radicals, into repairable and harmless radical forms. Further, vitamin E has a long biological half-life and very few side effects, even when administered in high doses. When rats are subjected to chronic OS followed by a series of assessments of learning and memory in water and radial mazes, the young ones exhibit reduced memory almost equivalent to normal aged ones, and these results have been related to a late onset of apoptosis in the CA1 site of the HC at around 7 days following stress. However, the responses are reversed by vitamin E in the young and, more importantly, in the old[18]. Vitamin E effectively blocks hydrogen peroxide ($H_2O_2$) formation and prevents its cytotoxic effects[19]. Regular supplementation of vitamin E from 18 months of age in male rats and continued thereafter for 3 months reduces oxidative damage in the CC through an up-regulation of the antioxidant defense[20]. The cholinergic system has a prominent role in cognition, with the cholinergic neurons acting on the cortex and facilitating the release of ACh that excites the cortical neurons[21]. Further, vitamin E intervention on the cholinergic system and memory in rats has proved that the antioxidant in combination with nicotine or pilocarpine can induce potentiation and improve memory retention of certain learning skills, such as passive avoidance[22]. It is established that neurological symptoms, such as seizures, occurring in patients with type II hyperprolinemia[23] are due to tissue accumulation of proline. In animals that are pretreated with trolox, a water-soluble vitamin E, along with vitamin C, acts synergistically to reduce the inhibition of acetylcholine esterase (AChE) activity imposed by proline in the CC[24], a situation analogous to the inhibition of AChE by free radicals under oxidative stress[25] and aging[26]. However, a short-term vitamin E presupplementation for 4 weeks in 5- and 20-month-old C57BL6 mice was reported to be ineffective, both in terms of reducing oxidative damage in the CC of aged mice and ameliorating impoverished cognitive and motor function[27]. Studies of Dai and associates[28] on induced AD by abetal-1-40 revealed that vitamin E protects primary cortical neurons in culture from the toxic effects of induced $H_2O_2$. In humans, vitamin E obliterates the cognitive decline with age unlike vitamin C and carotene, which have no such effect[29]. On the other hand, Bjelakovic and coworkers[30] reported that treatment with vitamin E, either singly or combined, may increase disease prevalence and increased mortality in humans. Their suggestions are based on analyses of electronic databases of epidemiological findings on 68 randomized trials involving 232,606 random adults, with meta-analyses and multiregression for comparing the effect of covariates across the trials.

## EXERCISE AND THE AGING BRAIN

In general, exercise is reported to up-regulate protein synthesis, which in turn can improve cellular ability to remove damaged proteins postsynthetically by free radicals, with the capacity varying in the mitotic and nonmitotic cells[31]. In the last 2 decades, there has been a resurgence of studies on physical activity, attempting to study the effects of exercise on the aging brain. Most of the studies on enrichment of the CC through exercise are on the neurotransmitters and antioxidant defense systems to overcome OS. Rats exposed to enriched environments, such as wheels, ladders, and small mazes, exhibit significant cortical thickness, cortical weight, and AChE levels[14]. These changes in the brain in response to enrichment are positive, not only for a number of animal species, but also for humans. Further, physical activity is one of the components of enrichment in the animals, and active animals in a cage that interact with novel objects have increased expression of nerve growth factor (NGF) in the HC. An improved cognitive performance in older rats is related to a lowering of OS by regular physical training, such as swimming and treadmill exercise[32]. In a study conducted on rats trained to run on a treadmill for a term of 3 months, Ahmadi Asl and coworkers[33] observed that exercise had no effect on learning in older animals of 9 months of age unlike younger ones of 3 months. The disparity in the results on cognitive responses may be attributed not only to the type of exercise, short or long term, but also to its intensity. Compared to runners, swimmers are subjected to an extended training period since the rigors of swimming are uniformly distributed throughout the body. Since the response at the level of the animal is related to the gross response to exercise, swimming elicits greater benefits compared to running. Swimming is effective in up-regulating the antioxidant enzymes[34], such as superoxide dismutase (SOD), in the cortex of older animals, in scavenging superoxides, and improving the learning and memory tasks[35]. In a follow-up experiment, we noticed that mitochondria from the cortex of swim-trained older animals of 18 months of age contained increased levels of $H_2O_2$ as compared to those that were 4 months old, interestingly with a correlation between mitochondria $H_2O_2$ generation, Mn-SOD activity, and malondialdehyde (MDA) level. In these animals, a substantial increase in lipid and protein oxidation in the cortex was also observed. More importantly, exercise was also proven to be effective in evoking responses in this region when initiated as late as middle and old age[20]. In spite of the generation of free radicals such as $H_2O_2$, regular and moderate exercise exerts a hormetic effect and the mild stress is beneficial to overcome OS[36,37,38].

Animal studies also support the beneficial effects of physical exercise on memory[39] and, in fact, short-term memory can actually be increased in suitably trained rats[40]. Exercise can also increase the declining neuronal number in the HC of older rats[41] and reduce brain tissue loss in humans[42]. Regular exercise is beneficial to the brain in terms of increased capillarization, reduced oxidative damage, enhanced activities of proteosome and neprilysin, resulting in decreased accumulation of carbonyls, as well as improved memory[43]. When untrained rats are tested in a two-way shuttle box and multiple water-filled T-mazes, aged rats exhibit less avoidance than younger rats. Choline acetyltransferase (ChAT) that breaks down ACh is significantly increased in the aged rats after 6 days of training in a water-filled T-maze and this is unlike the younger rats. AChE activity in these studies was lower in the control older rats when compared to untrained younger rats. From these experiments, the researchers concluded that changes in the cholinergic enzymes in trained aged rats are some of the several compensatory mechanisms to facilitate neurotransmission under deficit cognitive situations[44]. Further, studies on freely moving rats demonstrated a lesser release of ACh in the 18-month-old than in the 2-month-old rat in the CC and HC, reflecting a reduction in ACh synthesis in these regions[45]. Our studies have demonstrated that swim-trained older rats have lowered AChE activity when supplemented with vitamin E compared to a set of untrained rats. The middle-aged trainees benefit in terms of better acquisition and retention with the supplement when tested in a T-maze. In the older, however, exercise by itself is effective in enhancing the learning ability. Further, these findings are analogous to the AChE inhibitors that are widely advocated to obtain positive responses in terms of boosting the ACh level in overcoming age-related memory deficits[46]. Besides αT, proanthocyanidin, yet another antioxidant, can effectively reduce OS in the cortex of adult rats and holds a promise for the future as a cognitive enhancer[47]. In essence, exercise can additionally enhance the capillary number in the cortex, providing increased circulation and, in turn, protecting against neuronal loss with age.

However, in the untrained or exhaustive exercise programs, exercise itself is a powerful generator of OS[48] and may adversely affect the brain, resulting in short-term impairment in cognitive ability.

## CONCLUSION

Studies on exercise and cognition in animals provide information that explains the basic mechanisms of cognition that may not be possible to obtain from humans due to several limitations and variations. In humans, however, cross-sectional studies have provided the strongest evidence in establishing the relationship between exercise and aging. From the above discussion, it follows that the key emphasis is on the benefits of enriching the brain through exercise and vitamin E that, if started as early as middle age, will protect regions such as the CC against oxidative insults that are set rolling at this age. There is no doubt that we need to define how much and how intense these exercise programs should be, especially in the elderly. Similarly, in using vitamin E as an enriching antioxidant for overcoming free radical–related OS and reduced cognitive ability with advanced age, it is imperative to define the level and duration of supplementation that are needed. Hence, exercise and vitamin E may still prove to be far more beneficial in contrast to pharmacotherapeutic administration of cholinomimetics in normal advanced aging to delay the onset of cognitive deficits. Although such agents have been effective in improving learning as well as memory even through early ages, a cause for concern is that these agents can cause a range of behavioral symptoms, such as overactivity, in the elderly. Further studies are necessary in order to evaluate the mechanisms of age-related synergistic effects of exercise and vitamin E enrichment in slowing the decline in learning and memory, which may be gender based.

## References

1. Hipkiss, A.R. (2006) Accumulation of altered proteins and ageing: causes and effects. *Exp. Gerontol.* **41**, 464–473.
2. Sasaki, T., Unno, K., Tahara, S., Shimada, A., Chiba, Y., Hoshino, M., and Kaneko, T. (2008) Age-related increase of superoxide generation in the brains of mammals and birds. *Aging Cell* **7**, 459–469.
3. Niño-Cabrera, H.G., Coli-Barenque, L., Avila-Costa, M.R., Espinosa-Villanueva, J., Fortoul, T.I., and Rivas-Arancibia, S. (2002) Differences between hippocampus and cerebral cortex in aged rats in an oxidative stress model. *Int. J. Neurosci.* **112,** 373–381.
4. Martínez-Romero, R., Cañuelo, A., Martínez-Lara, E., Hernández, R., Del Moral, M.L., Pedrosa, J.A., Peinado, M.A., and Siles, E. (2006) Aging affects but does not eliminate the enzymatic antioxidative response to hypoxia/reoxygenation in cerebral cortex. *Exp. Gerontol.* **41**, 25–31.
5. Peinado, M.A., del Moral, M.L., Esteban, F.J., Martínez-Lara, E., Siles, E., Jimènez, A., Hernández-Cobo, R., Blanco, S., Rodrigo, J., Pedrosa, J.A. (2000) Aging and neurodegeneration: molecular and cellular bases. *Rev. Neurol.* **31**, 1054–1065.
6. Kan, S., Asha Devi, S., and Kawashima, S. (1991) Effect of vitamin E on the accumulation of fluorescent material in cultured cerebral cortical cells of mice. *Exp. Gerontol.* **26**, 365–374.
7. Benavides, S.H., Monserrat, A.J., Farina, S., and Porta, E.A. (2002) Sequential histochemical studies of neuronal lipofuscin in human cerebral cortex from the first to the ninth decade of life. *Arch. Gerontol. Geriatr.* **34**, 219–231.
8. Anitha, V. and Asha Devi, S. (1997) Responses of aging brain to physical exercise: changes in the substrates of energy metabolism. *Ind. J. Exp. Biol.* **35**, 280–284.
9. Praticò, D., Clark, C.M., Liun, F., Rokach, J., Lee, V.M.-Y., and Trojanowski, J.Q. (2002) Increase of brain oxidative stress in mild cognitive impairment: a possible predictor of Alzheimer's disease. *Arch. Neurol.* **59**, 972–976.
10. Nunomura, A., Perry, G., Aliev, G., et al. (2001) Oxidative damage is the earliest event in Alzheimer's disease. *J. Neuropathol. Exp. Neurol.* **60,** 759–767.
11. Smith, T.D., Adams, M.M., Gallagher, M., Morrison, J.H., and Rapp. P.R. (2000) Circuit-specific alterations in hippocampal synaptophysin immunoreactivity predict spatial learning impairment in aged rats. *J. Neurosci.* **20**, 6587–6593.
12. Fukui, K., Onodera, K., Shinkai, T., Suzuki, S., and Urano, S. (2001) Impairment of learning and memory in rats caused by oxidative stress and aging, and changes in antioxidative defense systems. *Ann. N. Y. Acad. Sci.* **928**, 168–175.

13. Onodera, K., Omoi, N.O., Fukui, K., Hayasaka, T., Shinkai, T., Suzuki, T., Abe, K., and Urano, S. (2003) Oxidative damage of rat cerebral cortex and hippocampus, and changes in antioxidative defense systems caused by hyperoxia. *Free Radic. Res.* **37,** 367–372.
14. Diamond, M.C. (2001) Response of the brain to enrichment. *An. Acad. Bras. Ciênc.* **73**, 1–11.
15. Lockrow, J., Prakasam, A., Huang, P., Bimonte-Nelson, H., Sambamurti, K., and Granholm, A.C (2009) Cholinergic degeneration and memory loss delayed by vitamin E in a down syndrome mouse model. *Exp. Neurol.* **216**, 278–289.
16. Blacker, D. (2005) Mild cognitive impairment--no benefit from vitamin E, little from donepezil. *New Engl. J. Med.* **352,** 2439–2441.
17. Hosomi, A., Goto, K., and Kondo, H. (1998) Localization of α-tocopherol transfer protein in rat brain. *Neurosci. Lett.* **256**,159–165.
18. Fukui, K., Onodera, K., Hayasaka, T., Shinkai, T., Suzuki, S., Abe, K and Urano, S. (2002) Cognitive impairment of rats caused by oxidative stress and aging, and its prevention by vitamin E. *Ann. N. Y. Acad. Sci.* **959**, 275–284.
19. Behl, C., Davis, J.B., Lesley, R., and Schubert, D. (1994) $H_2O_2$ mediates amyloid beta protein toxicity. *Cell* **77**, 817–827.
20. Jolitha, A.B., Subramanyam, M.V.V., and Asha Devi, S. (2006) Modification by vitamin E and exercise of oxidative stress in regions of aging rat brain: studies on superoxide dismutase isoenzymes and protein oxidation status. *Exp. Gerontol.* **41**, 753–763.
21. Perry, E.K., Lee, M.L., Martin-Ruiz, C.M., Court, J.A., Volsen, S.G., et al. (2001) Cholinergic activity in autism; abnormalities in the cerebral cortrex and forebrain. *Am. J. Physiol*. **158**, 1058–1066.
22. Eidi, A., Eidi, M., Mahmoodi, G., and Oryan, S. (2006) Effect of vitamin E on memory retention in rats: possible involvement of cholinergic system. *Eur. Neuropsychopharmacol.* **16**, 101–106.
23. Phang, J.M., Hu, C.A., and Cand, V.D, (2001) Disorders of proline and hydroxyproline metabolism. In *The Metabolic and Molecular Bases of Inherited Disease*, 8th ed., Scriver, C.R., Benauder, A.L., Sly, W.S., and Valle, D., Eds. McGraw-Hill, New York. pp.1821–1838.
24. Delwing, D., Chiarani, F., Delwing, D., Bavaresco, C.S., Wannacher, C.M.S., Wajner, M., and Wyse, A.T.S. (2003) Proline reduces acetylcholinesterase activity in cerebral cortex of rats. *Metab. Brain Dis.* **18**, 79–86.
25. Tsakiris, S., Angelogianni, P., Schulpis, K.H., and Stavridis, J.C. (2000) Protective effects of L-phenylalanine on rat brain acetylcholinesterase inhibition induced by free radicals. *Clin. Biochem.* **33,** 103–116.
26. Asha Devi, S. and Kiran, R.T. (2004) Regional responses of antioxidant system in exercise training and dietary vitamin E in aging rat brain. *Neurobiol. Aging* **25**, 501–508.
27. Sumien, N., Heinrich, K.R., Sohal, R.S., and Forsterm, M.J. (2004) Short-term vitamin E intake falls to improve cognitive neuropsychomotor performance of aged mice. *Free Radic. Biol. Med.* **36**, 1424–1433.
28. Dai, X., Sun, Y., and Jiang, Z. (2007) Protective effects of vitamin E against oxidative damage induced by Abetal-40Cu(II) complexes. *Acta Biochem. Biophys. Sin. (Shangai)* **39**, 123–130.
29. Morris, C.M., Denis, A., Evans, M.D., Julia, L.B., Christine, C.T., and Robert, W. (2002) Vitamin E and cognitive decline in older persons. *Arch. Neurol.* **59**, 1125–1132.
30. Bjelakovic, G., Nikolova, D., Gluud, L.L., Simonetti, R.G., and Gluud, C. (2007) Mortality in randomized trials of antioxidant supplements for primary and secondary prevention. *JAMA* **297**, 842–857.
31. Hipkiss, A.R. (2008) Energy metabolism, altered proteins, sirtuins and aging: converging mechanisms? *Biogerontology* **9**, 49–55.
32. Goto, S., Naito, H., Kaneko, T., Chung, H.Y., and Radak, Z. (2007) Hormetic effects of exercise in aging: correlation with oxidative stress. *Appl. Physiol. Nutr. Metab.* **32**, 948–953.
33. Ahmadi Asl, N., Sheikhzade, F., Torchi, M., Roshangar, L., and Khamnei, S. (2008) Long-term regular exercise promotes memory and learning in young but not in older rats. *Pathophysiology* **15**, 9–12.
34. Radak, S., Kaneko, T., Tahara, S., Nakamato, H., Puesok, J., and Sasvari, M. (2001) Regular exercise improves cognitive function and decreases oxidative damage in rat brain. *Neurochem. Int*. **38**, 17–23.
35. Asha Devi, S. and Prathima, S. (2005) Synergistic effects of physical exercise and antioxidants in aging mammalian brain. In *Molecular and Cellular Neurobiology.* Thakur, M.K. and Prasad, S., Eds. Narosa Publishing House, New Delhi. pp. 123–133.
36. Radak, Z., Chng, H.Y., Koltai, E., Taylor, A.W., and Goto, S. (2008) Exercise, oxidative stress and hormesis. *Ageing Res. Rev*. **7**, 34–42.
37. Rattan, S.I. (2008) Increased molecular damage and heterogeneity as the basis of ageing. *Biol. Chem*. **389**, 267–272.
38. Ji, L.L. (2007) Physical activity: a strong stimulant for hormesis during aging. In *Mild Stress and Healthy Aging; Applying Hormesis*. Le Bourg, E. and Rattan, S., Eds. SPI Publishing. pp. 97–114.
39. Cotman, C.W. and Berchtold, N.C. (2002) Exercise: a behavioral intervention to enhance brain health and plasticity. *Trends Neurosci*. **25**, 295–300.
40. Uysal, N., Tugyan, K., Kayatekin, B.M., Acikgoz, O., Bagriyanik, H.A., Gonenc, S., Ozdemir, D., Aksu, I., Topeu, A., and Semin, I. (2005) The effects of regular aerobic exercise in adolescent period on hippocampal neuron density, apoptosis and spatial memory. *Neurosci. Lett.* **383**, 241–245.
41. Kima, Y.P., Kim, H., Shin, M.S., Chang, H.K., Jaung, M.H., Shin, M.C., Lee, S.J., Lee, H.H., Yoon, J.H., Jeong, I.G.C.J., and Jeong, C.J. (2004) Age-dependence of the effect of treadmill exercise on cell proliferation in the dentate gyrus of rats. *Neurosci. Lett*. **355**, 152–154.

42. Colcombe, S.J., Erickson, N., Raz, K.I., Webb, A.G., Cohen, N.J., McAuley, E., and Kramer, A.F. (2003) Aerobic exercise reduces brain tissue loss in aging humans. *J. Gerontol. A Biol. Sci. Med. Sci*. **58**, 176–180.
43. Radak, Z., Kumagai, S., Taylor, R.W., Naito, H., and Goto, S. (2007) Effects of exercise on brain function: role of free radicals. *Appl. Physiol. Nutr. Metab*. **32**, 942–946.
44. Nakamura, S. and Ishihara, T. (1989) Region selective increase in activities of CNS cholinergic marker enzymes during learning of memory tasks in aged rats. *Pharmacol. Biochem. Behav*. **34**, 805–810.
45. Wu, M., Shanabrough, M., Leranth, C., and Alreja, M. (2000) Cholinergic excitation of septohippocampal GABA but not cholinergic neurons: implications for learning and memory. *J. Neurosci*. **265**, 676–679.
46. Jolitha, A.B., Subramanyam, M.V.V., and Asha Devi, S. (2009) Age-related responses of the rat cerebral cortex: influence of vitamin E and exercise on the cholinergic system. *Biogerontology* **10**, 53–63.
47. Asha Devi, S., Jolitha, A.B., and Ishii, N. (2006) Grape seed proanthocyanidin extract (GSPE) and antioxidant defense in the brain of adult rats. *Med. Sci. Monit.* **12**, BR124–129.
48. Gomez-Cabrera, M.C., Domenech, E., and Viña, J. (2008) Moderate exercise is an antioxidant upregulation of antioxidant genes by training. *Free Radic. Biol. Med.* **15,** 126–131.
